# Microstructures, mechanical behavior and strengthening mechanism of TiSiCN nanocomposite films

**DOI:** 10.1038/s41598-017-02186-1

**Published:** 2017-05-18

**Authors:** Wei Li, Ping Liu, Zenghui Xue, Fengcang Ma, Ke Zhang, Xiaohong Chen, Rui Feng, Peter K. Liaw

**Affiliations:** 1 School of Materials Science and Engineering, University of Shanghai for Science and Technology, Shanghai, 200093 PR China; 2 State Key Laboratory of Superhard Materials, Jilin University, Changchun, 130012 PR China; 3 Department of Materials Science and Engineering, The University of Tennessee, Knoxville, TN 37996 USA

## Abstract

Currently, the arguments have existed in the strengthening mechanism and microstructural model of the nanocomposite film due to lack of the convincible experimental evidences. In this investigation, the quarternary TiSiCN nanocomposite films with the different C and Si contents are synthesized by the reactive-magnetron-sputtering technique. The TiSiCN film is characterized as the nanocomposite structure with the TiN nanocrystallites surrounded by the (Si_3_N_4_ + C + CN_x_) interface phase. When the C/Si content ratio is 2:2, the TiSiCN nanocomposite film is remarkably strengthened with the maximal hardness and elastic modulus of 46.1 GPa and 425 GPa, respectively. Meanwhile, the (Si_3_N_4_ + C + CN_x_) interfaces exhibit as a crystallized form, which can coordinate the growth misorientations and maintain the coherently epitaxial growth between the TiN nanocrystallites and interfaces. Through the high-resolution transmission electron microscopy (HRTEM) observations, this investigation firstly provides the direct experimental evidence for the crystallized feature of the interfaces when the TiSiCN nanocomposite film is strengthened, suggesting that the strengthening effect of the TiSiCN nanocomposite film can be attributed to the coherent-interface strengthening mechanism, which is expressed as the “nc-TiN/c-Si_3_N_4_/c-C/c-CN_x_” model.

## Introduction

The nanocomposite films, as a new type of the superhard film material (≥40 GPa), successfully improve the hardness, wear resistance, thermal stability, and other properties, which have been extensively studied in the past decades^1–3^. Such films are constituted of the nanocrystallites (≤10 nm) of transition metal nitrides, carbides, or borides surrounded by the interface layers. As a typical representative among these films, TiSiN was firstly synthesized using the plasma enhanced chemical vapor deposition (PECVD) by Li and his coworkers in 1992^4^ and exhibits high hardness ranging from 32 GPa to 105 GPa deposited by the different methods^5–8^, which has attracted much attention in the field of material-surface engineering.

In the recent years, the carbon-incorporated quarternary TiSiCN nanocomposite films have been brought forward in order to further elevate the hardness and improve the friction and tribological properties. The TiSiCN nanocomposite films with the appealing mechanical properties have been successfully synthesized by chemical vapor deposition (CVD)^9, 10^, magnetron sputtering^11^ and the hybrid methods^12, 13^. The microstructure and mechanical properties of the TiSiCN films were shown to sensitively depend on the Si and C contents in the films^9–13^. As for the strengthening mechanism, many researchers were attributed the strengthening effect of the TiSiCN nanocomposite film to a “nc-nanocrystallite/a-interface” model^9–16^, in which the equiaxed nanocrystallites (nc-nanocrystallite) were embedded in the amorphous interfaces (a-interface). This model originates from the “nc-TiN/a-Si_3_N_4_” model proposed by Veprek in 1995 to describe the strengthening mechanism of the TiSiN nanocomposite film^17^. However, the “nc-nanocrystallite/a-interface” model is in dispute due to the lack of the sufficient and direct experimental evidence^18–20^, which mainly reflects whether the interfaces exhibit as an amorphous or a crystallized state. For example, Hultman *et al*. reported that the Si_3_N_4_ interface layer could be crystallized by the adjacent TiN nanocrystallites based on their ab initio calculations^18^. Unfortunately, they did not give direct experimental evidence.

To this end, the quarternary TiSiCN nanocomposite films with the different C and Si contents are synthesized by the reactive-magnetron-sputtering technique. The microstructure and behavior of the TiSiCN nanocomposite films with the different C and Si contents will be studied in the investigation. Special attention will be paid to the interface structure through the high-resolution transmission electron microscopy (HRTEM) observations when the film is strengthened, with an expectation of providing the direct experimental evidence for revealing the intrinsic strengthening mechanism of the TiSiCN nanocomposite film.

## Results

### Composition and microstructures

In consideration of the possible difference in sputtering rates of C and Si, the chemical compositions of five TiSiCN films deposited from TiSiC compound targets with different C/Si ratios are characterized by energy dispersive spectroscopy (EDS), as shown in Table 1. It can be seen that with the increase of the C/Si ratio in the TiSiC target, the C content of the TiSiCN film constantly ascends, while the Si content continually decreases. The N contents for all the TiSiCN films are close to 50 atomic percent (at.%), suggesting that the films are formed as the saturated nitrides. The (C + Si) contents of the TiSiCN films do not change much among the TiSiCN films, which are in a small range from 9.65 at.% to 12.17 at.%. The C/Si ratios in TiSiCN films are basically consistent with those in the TiSiC targets.

**Table 1:** Compositions of as-deposited TiSiCN films, as determined by EDS.

C/Si ratio in target	Elemental composition (at. %)				C/Si ratio in film	$\frac{({\boldsymbol{C}}{\boldsymbol{+}}{\boldsymbol{Si}}){\boldsymbol{ \% }}}{({\boldsymbol{Ti}}{\boldsymbol{+}}{\boldsymbol{C}}{\boldsymbol{+}}{\boldsymbol{Si}}{\boldsymbol{+}}{\boldsymbol{N}}){\boldsymbol{ \% }}}$
	Ti	C	Si	N		
0:4	37.67	—	9.65	52.68	0	09.65%
1:3	38.78	3.09	8.29	49.84	0.373	11.38%
2:2	35.85	5.23	5.74	53.18	0.911	10.97%
3:1	36.10	8.94	3.23	51.73	2.768	12.17%
4:0	35.39	11.40	—	53.21	—	11.40%

**Figure 1:**
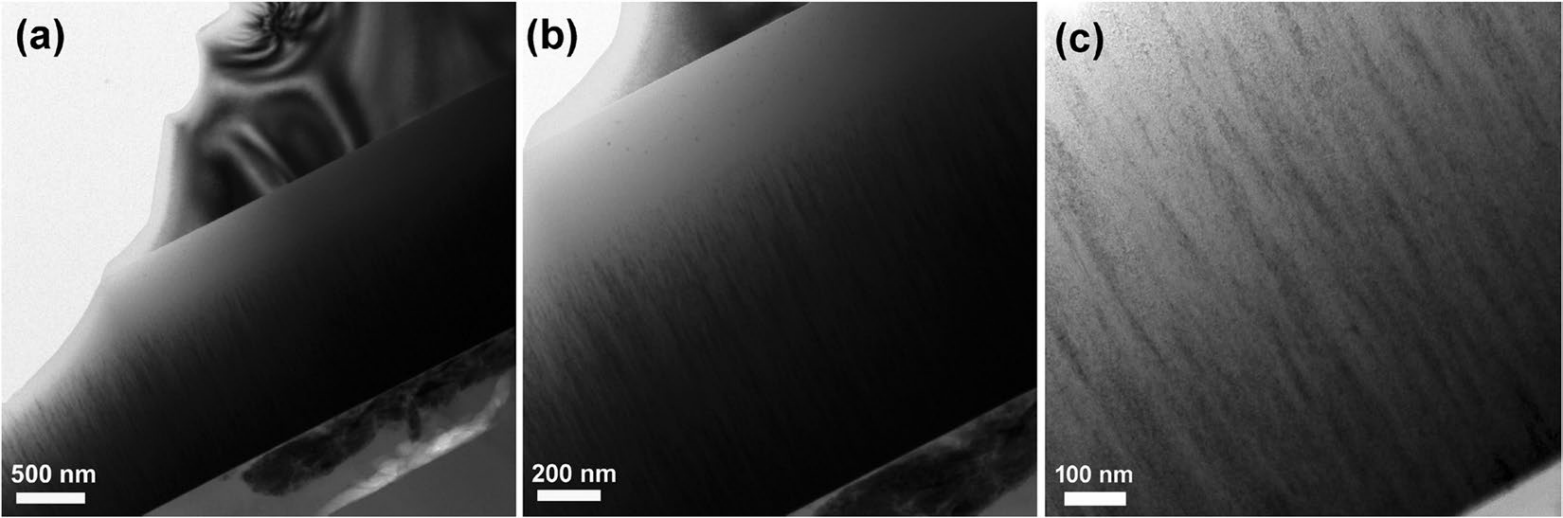
Cross-sectional TEM images of the TiSiCN film with the C/Si content ratio of 2:2. (**a**) low-magnification, (**b**) medium-magnification, and (**c**) high-magnification. The TiSiCN film exhibits elongated fibrous morphology, with the fiber width of about 20 nm–50 nm.

Figure 1 shows the typical cross-sectional TEM images of the TiSiCN film with the C/Si content ratio of 2:2. It is clear from the TEM observations that the TiSiCN film presents the dense and compact structure. Based on the low-magnification image of Fig. 1(a), it can be seen that the thickness of the TiSiCN film is about 2 μm. It is worth pointing out that the incomplete surface is caused by the ion bombardment during the TEM specimen preparation. The magnified Fig. 1(b) and (c) show that the TiSiCN film exhibits elongated fibrous morphology, with the fiber width of about 20 nm–50 nm. This width is much smaller than that of 50 nm–100 nm of the TiSiN nanocomposite film (with the C/Si content ratio of 0:4^21^), suggesting that the addition of C can effectively refine the microstructure of the TiSiCN film.

**Figure 2:**
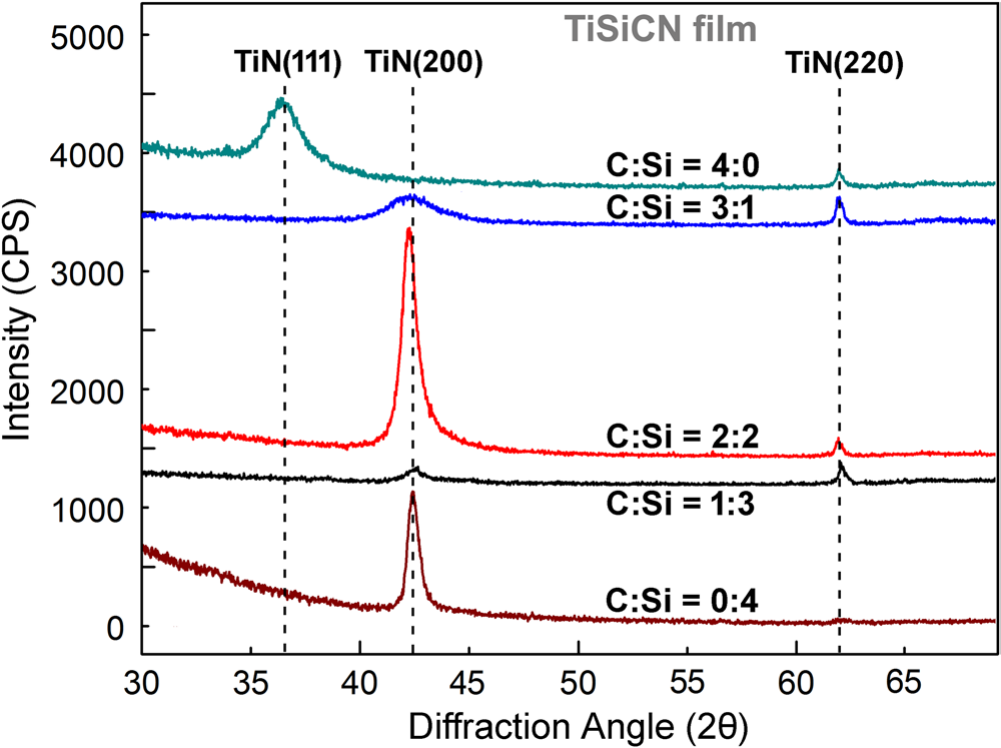
XRD patterns of the TiSiCN nanocomposite films with the different C/Si content ratios. The diffraction peaks refer to the face-centered cubic structured TiN phase. No other diffraction peaks from crystalline phases can be detected. The TiSiCN film with the C/Si content ratio of 2:2 presents the highest diffraction peak intensity.

The X-ray diffraction (XRD) patterns of the TiSiCN nanocomposite films with the different C/Si ratios are presented in Fig. 2. The diffraction peaks located at 36.7°, 42.5°, and 61.8° can be observed, which refer to (111), (200), and (220) crystal planes of the face-centered cubic (fcc) structured TiN phase, respectively. Some reports suggested that these diffraction peaks could belong to the fcc-TiCN^9–11, 14, 16^. Since the X-ray diffraction features of TiN are very similar to TiCN, the phase structure of the TiSiCN film needs to be further identified by the X-ray photoelectron spectroscopy (XPS) results, which will be presented later. No other diffraction peaks from crystalline phases, such as Si_3_N_4_ and C, can be detected in the XRD patterns.

When the C/Si content ratio is 0:4 (no C element is added in the TiSiN film), the film exhibits a pronounced preferential (200) orientation. As the C/Si content ratio increases to 1:3, the intensity of the (200) diffraction peak descends, suggesting that the crystallization degree of the film decreases. When the C/Si content ratio further increases to 3:1, the intensity of the (200) diffraction peak firstly increases and then decreases, and reaches the maximum value with the C/Si content ratio of 2:2, implying that the TiSiCN film exhibits the highest crystallization degree. Using the Scherrer formula, the crystallite sizes for TiSiCN nanocomposite films with the C/Si content ratios of 0:4 and 2:2 can be calculated from the (200) peaks as about 46.78 nm and 21.45 nm, respectively, which is basically in accordance with the TEM observations.

As the Si element is fully replaced by the C element (the C/Si content ratio is 4:0), the preferred orientation of the film changes from (200) to (111). The absence of Si leads to the disappearance of the nanocomposite structure and formation of the columnar structure within the TiCN film^22^. Without the hindering effect of the interface within the nanocomposite film, the TiCN film is inclined to grow along the (111) crystal plane, which has the lowest energy in the fcc structure^23^.

**Figure 3:**
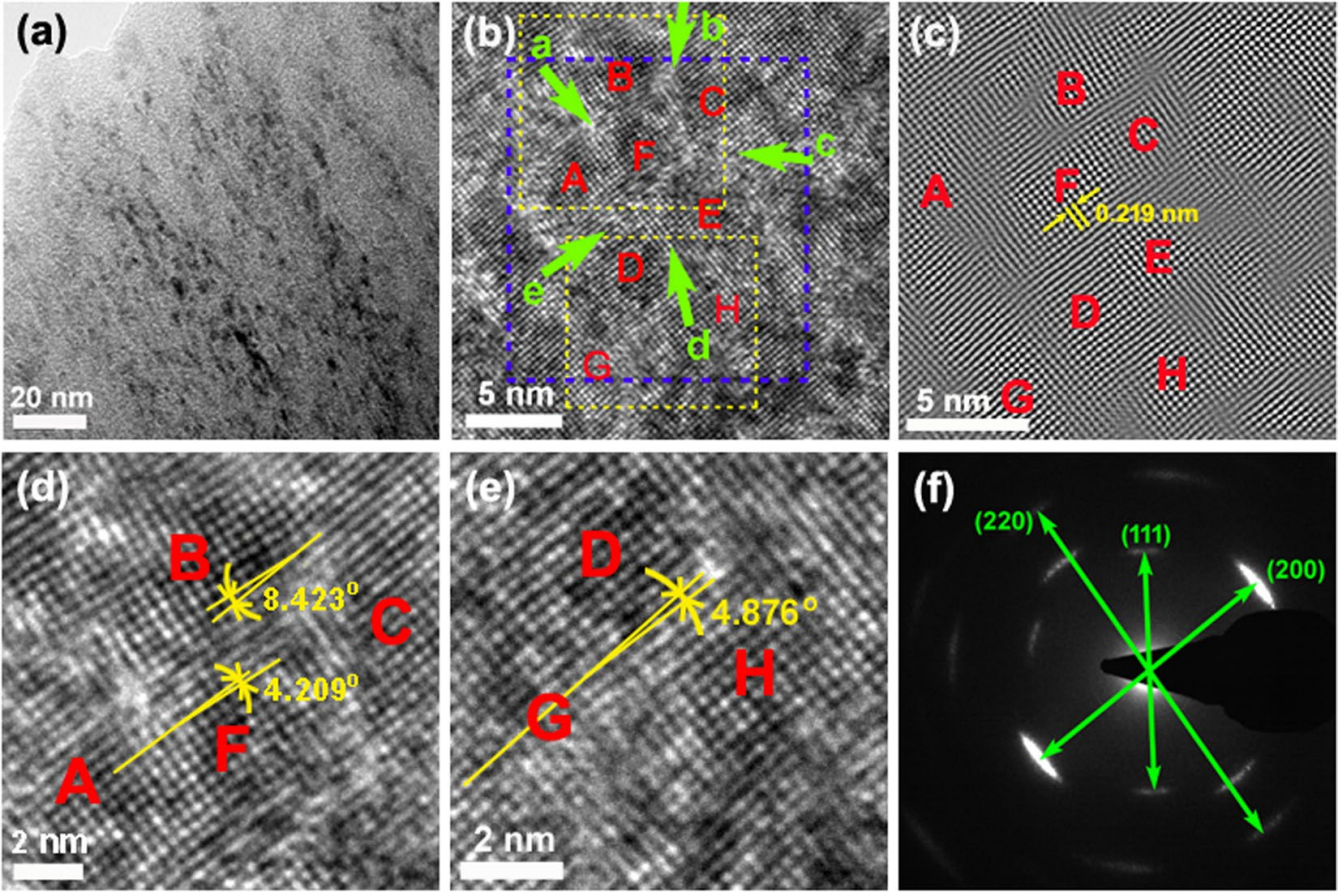
Cross-sectional HRTEM images of the TiSiCN nanocomposite film with the C/Si content ratio of 2:2. (**a**) Low magnification. There exist many nano-sized equiaxed grains with the dark contrast within the elongated grains. (**b**) High magnification. The nanocomposite structure is created with the equiaxed nanocrystallites surrounded by the interfaces. The epitaxial growth structure between the nanocrystallites and interfaces is basically revealed. (**c**) Inverse fast Fourier transformation micrograph of the area marked by the blue frame in (**b**). (**d**) and (**e**) the magnified images from the upper and lower yellow dashed line areas in (**b**), indicating that the small growth misorientations can be found between the adjacent nanocrystallites. (**f**) Selected area electron diffraction patterns.

Due to the presentation of the highest crystallization degree, the cross-sectional microstructures of the TiSiCN film with the C/Si content ratios of 2:2 are observed through HRTEM, as shown in Fig. 3. From the low-magnification image of Fig. 3(a), it is clear that, within the elongated grains, there exist many nano-sized equiaxed grains with the dark contrast. From the high-magnification image of Fig. 3(b), the equiaxed nanocrystallites denoted by *A*, *B*, *C*, *D*, *E*, *F*, *G*, and *H* can be observed with an average size of 4 nm–8 nm. Between the adjacent nanocrystallites, there exist the interfaces with the bright contrast marked by *a*, *b*, *c*, *d*, and *e* green arrows with an average thickness of about 0.5 nm–2 nm. Obviously, the nanocomposite structure is created within the TiSiCN film with the equiaxed nanocrystallites surrounded by the interfaces. It can also be seen that the lattice fringes can continuously go across several nanocrystallites and interfaces, indicating that the interfaces exist as a crystallized state, rather than an amorphous state suggested by some researches^9–16^. Furthermore, the epitaxial growth structure between the nanocrystallites and interfaces can be basically revealed.

Fig. 3(c) is an inverse fast Fourier transformation (IFFT) micrograph of the area marked by the blue frame in Fig. 3(b). It can be clearly seen that the nanocrystallite areas present the comparatively perfect lattice, while the interface areas show the distortion of lattice. Based on the nanocrystallite area, an interplanar spacing of *d* = 0.219 nm was measured, which agrees well with that of the fcc TiN (200) (JCPDS No. 38-1420 (2004)), suggesting that the nanocrystallites are constituted of TiN. Meanwhile, the small growth misorientations can be found between the adjacent nanocrystallites, such as *A* and *B*, *B* and *F*, *D* and *G*, *C* and *E*. As a result, it can be concluded that the interfaces can help to coordinate the misorientations between the adjacent TiN nanocrystallites.

Fig. 3(d) and (e) are the magnified images from the upper and lower yellow dashed line areas in Fig. 3(b), respectively. It can be seen from Fig. 3(d) that there exist the misorientations with 4.209° between nanocrystallites *A* and *F*, with 8.423° between nanocrystallites *B* and *C*. Fig. 3(e) also indicates that the nanocrystallite *D* has the misorientation of 4.876° with the nanocrystallite *G*. Nevertheless, these nanocrystallites can basically maintain the epitaxial growth structure through the buffering and coordination functions of the interfaces. Therefore, the crystallized interfaces can play a role in coordinating the misorientations between the adjacent nanocrystallites and maintaining the coherently-epitaxial growth between nanocrystallites, which improves the crystallization degree of the film. The selected area electron diffraction (SAED) patterns shown in Fig. 3(f) were obtained with electron beam of about 180 nm in diameter, indicating that the TiSiCN film presents a fcc structure with the (200) preferred orientation, which is consistent with the XRD results. The elongation of the diffraction spots can be attributed to the strong preferential (200) orientation.

**Figure 4:**
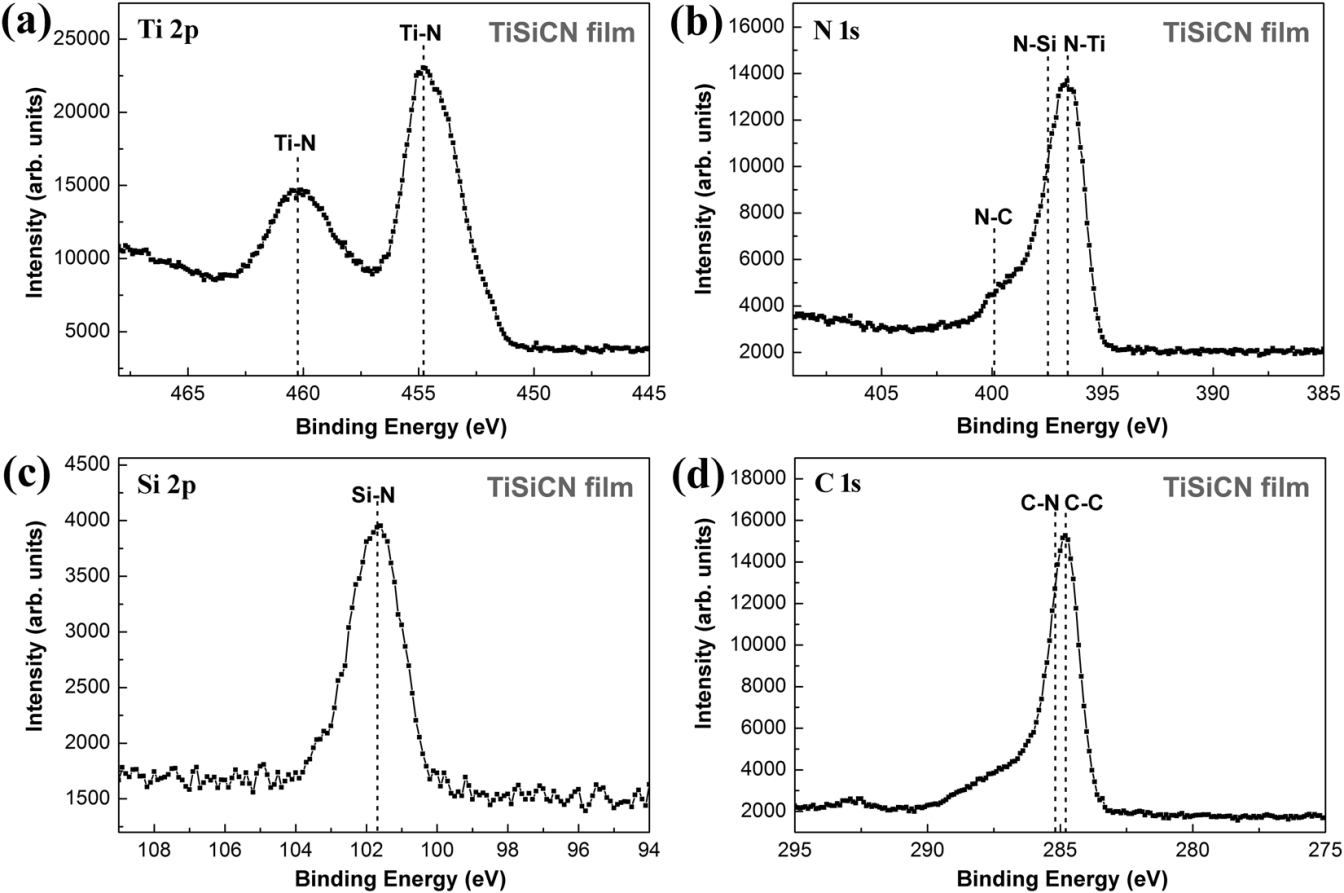
XPS spectra of the TiSiCN nanocomposite film with the C/Si content ratio of 2:2. (**a**) Ti 2p, (**b**) N 1 s, (**c**) Si 2p, and (**d**) C 1 s. The TiN, Si_3_N_4_, C, and CN_x_ can be identified. No information of TiC can be detected in the spectra.

In order to identify the phase structure of the nanocrystallites and interfaces, the XPS analysis has been carried out for the TiSiCN nanocomposite films with the C/Si content ratio of 2:2. The typical Ti 2p, N 1 s, Si 2p, and C 1 s XPS spectra are presented in Fig. 4. The Ti 2p spectrum in the Fig. 4(a) shows the characteristic doublet of Ti, which are attributed to the 2p_3/2_ and 2p_1/2_ peaks, respectively. The TiN phase can be identified by the 2p_3/2_ peak at 454.8 eV and the 2p_1/2_ peak at 460.2 eV^24, 25^. The N 1 s peak is shown in the Fig. 4(b). The three peaks corresponding to 396.8 eV, 397.5 eVand 399.8 eV are in agreement with the binding energies of TiN^26^, Si_3_N_4_^27^, and CN_x_^28^, respectively. Figure 4(c) indicates that the peak observed at 101.7 eV in the Si 2p spectrum belongs to Si_3_N_4_^29^. The C 1 s peak in the Fig. 4(d) can be resolved into a major component at 284.8 eV consistent with C-C^30^ and a smaller component at 285.2 eV from C-N^31^. No information of C-Ti (282.2 eV^32^) can be observed in the C 1 s spectrum.

According to the results from XRD, HRTEM, and XPS studies, it is reasonable to believe that the quarternary TiSiCN film with the C/Si content ratio of 2:2 is characterized as the nanocomposite structure with the TiN nanocrystallites surrounded by the interface phase. The interface phase is constituted of Si_3_N_4_, C, and CN_x_. With the C/Si content ratio of 2:2, the interfaces consisted of Si_3_N_4_, C, and CN_x_ cannot be present in the amorphous state, as some researchers widely suggested^9–16^, but exhibit as a crystallized form, which can coordinate the growth misorientations between the adjacent TiN nanocrystallites and help to maintain the coherently-epitaxial growth between the TiN nanocrystallites and interfaces.

### Hardness and elastic modulus

**Figure 5:**
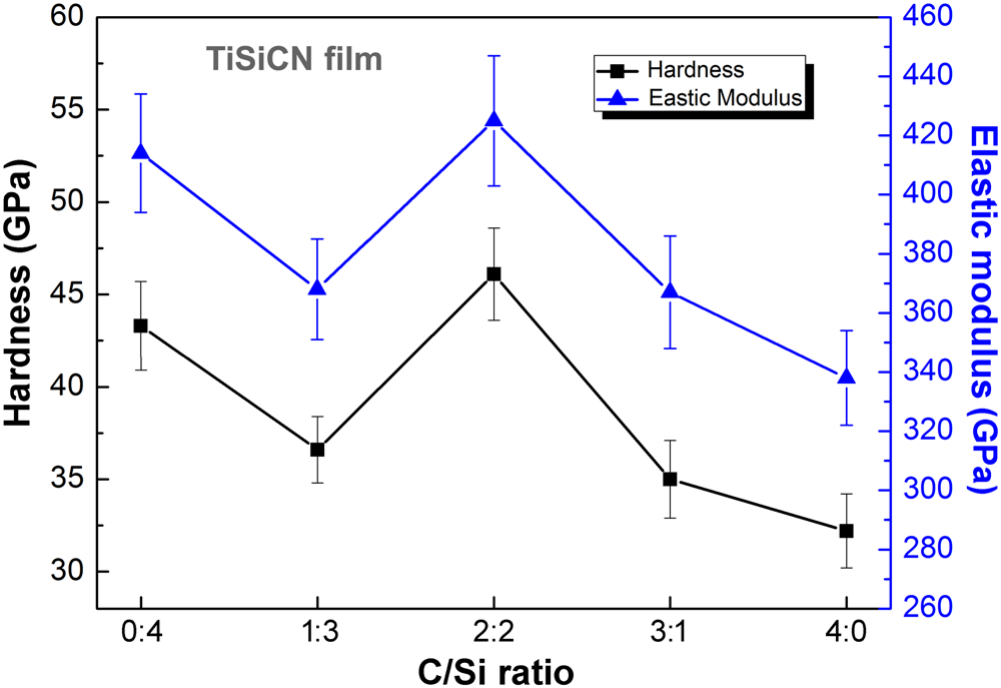
Variation of the hardness and elastic modulus of the TiSiCN nanocomposite films with the change of the C/Si content ratio. The hardness and elastic modulus reach the maximum values with the C/Si content ratio of 2:2.

The hardness (*H*) and elastic modulus (*E*) of the TiSiCN nanocomposite films with the change of the C/Si content ratio are shown in Fig. 5. As the C/Si content ratio is 0:4, namely, no C is added in the film, the TiSiN nanocomposite film achieves the high hardness and elastic modulus values of 43.3 GPa and 414 GPa, respectively. When the C/Si content ratio increases to 1:3, the initial addition of the C element leads to the decrease of the hardness and elastic modulus of the film. As the C/Si content ratio rises to 2:2, however, the hardness and elastic modulus renewedly improve and reach the maximum values of 46.1 GPa and 425 GPa, respectively. With the further increase of C content, the hardness and elastic modulus gradually decrease, which drop to 32.2 GPa and 338 GPa for the TiCN film, respectively.

The TiSiN film is well known as the nanocomposite structure with the TiN nanocrystallites encapsulated by the Si_3_N_4_ interface phase, leading to the high hardness and elastic modulus^5–7^. According to our previous investigation on the TiSiN nanocomposite film^21^, the Si_3_N_4_ interfacial phase could exist as crystallized state and grow epitaxially with the adjacent TiN nanocrystallites, which could block the dislocation movements and strengthen the TiSiN nanocomposite film. With the C/Si content ratio of 1:3, according to the XRD results in Fig. 2, the crystallization degree of the film remarkably decreases, suggesting that the initial addition of the C element disturbs the nanocomposite structure within the film and relieves the strengthening effect from the nanocomposite structure, and therefore, decreases the mechanical properties of the film.

When the C/Si content ratio rises to 2:2, based on the above analysis, together with the Si_3_N_4_, the increased content of C can also exist at the interface. The crystallized interfaces consisted of Si_3_N_4_, C, and CN_x_ can coordinate the growth misorientations between the adjacent TiN nanocrystallites and maintain the coherently-epitaxial growth between the TiN nanocrystallites and interfaces. These coherent interfaces can effectively stabilize the nanocomposite structure and restrain the generation and movement of dislocations^33, 34^, leading to the remarkable strengthening effect and improvement of the mechanical properties. As a result, the high hardness and elastic modulus of 46.1 GPa and 425 GPa can be achieved, respectively.

As the C/Si content ratio further increases, the C content raises, while the Si content reduces. At the same time, the Si_3_N_4_ interface phase accordingly decreases. It is worth noting that the nanocomposite structure of the TiSiCN film originates from the thermodynamic incompatibility between the TiN and Si_3_N_4_. The reduction of the Si_3_N_4_ can weaken the nanocomposite structure and thus the strengthening effect resulted from it. Consequently, the hardness and elastic modulus remarkably decrease. Especially, when the C/Si content ratio rises to 4:0, no Si_3_N_4_ phase exists in the film, which can divide the TiN crystals. As a result, the nanocomposite structure disappears within the TiCN film, leading to the low hardness and elastic modulus of 32.2 GPa and 338 GPa, respectively.

## Discussion

### Existence form of C element

Some studies reported that the C element could combine with Ti and N elements to form TiC and/or TiCN phases during the synthesis of the TiSiCN film^9–11, 14, 16^. However, in this investigation, according to the XPS spectra, only C-C and C-N bonds are observed in C 1 s peak. Furthermore, no C-Ti bond is found in both Ti 2p and C 1 s peaks. These results suggest that the C element only exists in the form of the C and CN_x_ phases, and no TiC phase creates during the deposition. It is noted that the previous studies which reported to detect the TiC and/or TiCN phases synthesized the TiSiCN films by CVD or other plasma enhanced techniques^9–11, 14, 16^. In this investigation, the TiSiCN films are prepared by reactive magnetron sputtering techniques. Therefore, it is believed that the absence of the TiC and/or TiCN phases may be attributed to the lower activities of Ti and C from the solid TiSiC compound targets in this investigation, relative to the gas sources used in the CVD techniques or other plasma enhanced techniques.

**Figure 6:**
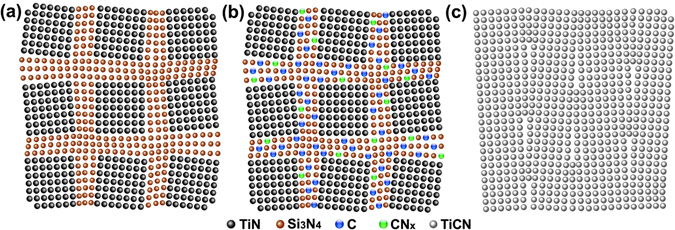
Schematic illustration of microstructural evolution of the TiSiCN nanocomposite film with the different C/Si content ratios. (**a**) 0:4. The Si_3_N_4_ interfaces surround the TiN nanocrystallites. (**b**) 2:2. The TiN nanocrystallites are surrounded by the Si_3_N_4_/C/CN_x_ interfaces, which present a crystallized form and coordinate the coherently-epitaxial growth between the TiN nanocrystallites and interfaces. (**c**) 4:0. The nanocomposite structure disappears and the columnar structure forms.

The C and CN_x_ can hardly dissolve into the TiN nanocrystallites, but only locate at the interfaces, which combine with Si_3_N_4_ to form the Si_3_N_4_/C/CN_x_ interface phase. The schematic illustration of microstructural evolution of the TiSiCN nanocomposite film with the different C/Si content ratios is shown in Fig. 6. When the C/Si content ratio is 0:4, the Si_3_N_4_ interfaces surround the TiN nanocrystallites and grow epitaxially with them, as suggested in our previous investigation^21^ and shown in Fig. 6(a). As the C/Si content ratio increases to 2:2, the TiN nanocrystallites are surrounded by the Si_3_N_4_/C/CN_x_ interfaces, which present a crystallized form and coordinate the growth misorientations between the adjacent TiN nanocrystallites, leading to the coherently-epitaxial growth between the TiN nanocrystallites and interfaces, as illustrated in Fig. 6(b). This microstructural model of the TiSiCN nanocomposite film can be described as nc-TiN/c-Si_3_N_4_/c-C/c-CN_x_. When the C/Si content ratio further rises to 4:0, the nanocomposite structure disappears due to the absence of Si_3_N_4_, resulting in the formation of columnar structure within the TiCN film^22^, as presented in Fig. 6(c).

Comparatively, based on the HRTEM observations in Fig. 3, the average thickness of the interface is about 0.5 nm–2 nm, much larger than that (0.5 nm–0.7 nm) of the TiSiN nanocomposite film (the C/Si content ratio is 0:4)^21^. The thickening of the interface can lead to the improvement of the interface toughness, resulting from the presence of C and CN_x_. For the TiSiN nanocomposite film, the interface only consists of Si_3_N_4_, while the interface of the TiSiCN nanocomposite film is composed of Si_3_N_4_, C and CN_x_. The combination of C and CN_x_ contributes to making the interface more flexible, which can more effectively coordinate the growth misorientations between the TiN nanocrystallites and maintain the epitaxial growth. Moreover, the presence of C and CN_x_ at the interface helps to reduce the friction by the formation of the graphite-like C lubricating layer, which can improve the friction and tribological properties^35, 36^.

### Questions about the “nc-nanocrystallite/a-interface” model

Some researchers believed that the microstructural strengthening model of the TiSiCN nanocomposite film can be depicted as the nc-TiN or TiCN nanocrystallite/a-interface^9–16^. The identification of the amorphous state for the interfaces of the TiSiCN nanocomposite film mainly comes from their XRD and XPS results in these reports. On one hand, no diffraction peaks of the crystallized interface phases, such as Si_3_N_4_, C, and CN_x_, were detected in the XRD patterns. On the other hand, the Si-N, C-C, and C-N bonds were found in the XPS spectrum. Therefore, the interfaces within the TiSiCN film were identified as an amorphous state.

However, the absence of the crystallized Si_3_N_4_, C, and CN_x_ interface phases in the XRD patterns can also be explained by their low contents and other reasons. It is worth pointing out that in the investigations of the crystalline/amorphous nanomultilayered films, such as CrAlN/Si_3_N_4_^37^ and TiN/SiC^38^, the Si_3_N_4_ and SiC amorphous layers can be crystallized when their layer thicknesses are small enough (less than or about 1 nm) and grow epitaxially with the CrAlN and TiN layers. In such a case, the crystallized Si_3_N_4_ and SiC phases cannot be detected in the XRD and even SAED patterns^37–39^. Therefore, the identification of the amorphous characteristic for the interfaces within the TiSiCN nanocomposite film deserves to be further deliberated.

Through the HRTEM observations, this investigation firstly provides the direct experimental evidence for the crystallized feature of the interfaces when the TiSiCN nanocomposite film presents the strengthening effect, suggesting that the nc-nanocrystallite/a-interface model cannot be used to describe the strengthening mechanism of the TiSiCN nanocomposite film. The interface can not only present the crystallized state between the TiN nanocrystallites, but also coordinate the misorientations of the adjacent TiN nanocrystallites and maintain the coherently-epitaxial growth between nanocrystallites and interfaces, which can improve the crystallization degree and strengthen the film through the other mechanism, that is, the coherent-interface strengthening mechanism.

### Coherent-interface-based strengthening mechanism

According to the modulus-difference theory proposed by Koehler^40^, when the dislocations came across the coherent interface, they would be blocked at interfaces by the forces generated from two materials with different shear moduli. The larger the difference between the shear moduli was, the more remarkable of the strengthening effect was. The TiN nanocrystallites and Si_3_N_4_/C/CN_x_ interfaces have totally different shear moduli. Therefore, the TiSiCN nanocomposite film can be effectively strengthened. Based on the modulus-difference strengthening model^41^, the hardness increment (*ΔH*_*1*_) relative to their constituent with relatively-lower hardness (SiCN) can be calculated as:

1 $$\frac{3(1-\nu )({G}_{A}-{G}_{B})\sin \,\theta }{{\rm{m}}{\pi }^{2}}\,\le \,{\rm{\Delta }}{H}_{1}\,\le \,\frac{3({G}_{A}-{G}_{B})\sin \,\theta }{{\rm{m}}{\pi }^{2}}$$

where *ν* is the Poisson’s ratio, taking 0.25; *θ* is the angle between the interface and the glide plane of the crystal with a smaller elastic modulus, taking 45° in the investigation; *m* is the Taylor factor, taking 0.3 for the TiSiCN film^42^; *G*_*A*_ and *G*_*B*_ are the shear moduli of two phases, respectively. The shear modulus can be calculated as follows:

2 $${\rm{G}}=\frac{E}{2(1+v)}$$

where *E* is the elastic modulus. It is worth noting that the measured hardness and elastic moduli of the TiN film and SiCN film with the C/Si ratio of 2:2 deposited under the same conditions are *H*_*TiN*_ = 25.7 GPa, *E*_*TiN*_ = 284 GPa, and *H*_*SiCN*_ = 14.2 GPa, *E*_*SiCN*_ = 171 GPa, respectively. Using the measured elastic moduli values, the shear moduli of TiN and SiCN can be calculated as *G*_*TiN*_ = 113.6 GPa and *G*_*SiCN*_ = 68.4 GPa, respectively. Since *G*_*A*_ is larger than *G*_*B*_, *G*_*A*_ and *G*_*B*_ are therefore *G*_*TiN*_ and *G*_*SiCN*_, respectively. Substituting all these values in Eq. (1), the hardness increment (*ΔH*_*1*_) relative to SiCN can be calculated as 24.3 GPa–32.4 GPa.

Moreover, the TiN nanocrystallites and interfaces have different lattice parameters. Especially, the lattice parameter of interface is supposed to be larger than that of the TiN nanocrystallite due to the inclusion of Si_3_N_4_, C, and CN_x_ phases. Under the epitaxial growth structure, the interface with the larger lattice parameter is subjected to the compressive stress, while the TiN nanocrystallite with the smaller lattice parameter is supposed to endure the tensile stress, which can be verified by the phenomenon that the TiN (200) diffraction peak of the TiSiCN film with the C/Si content ratio of 2:2 shows a shift towards a low angle in Fig. 2. As a result, the compressive and tensile stress fields create within the TiSiCN nanocomposite film, which can block the dislocation motion and thus, strengthen the film based on the alternating-stress theory suggested by Kato^43^.

According to the alternating stress-field theory, the hardness increment (*ΔH*_*2*_) can be expressed as:

3 $$\Delta H\cong 3\sigma =\frac{\sqrt{6}}{2}A\varepsilon {E}_{{\rm{ave}}}$$

where *A* is amplitude influenced by the modulation period, and roughness and width of interfaces, taking 0.5 in this study^44^; *E*_*ave*_ is the weighted average modulus, and is approximately calculated as 261.4 GPa for the TiSiCN nanocomposite film with the C/Si content ratio of 2:2. *ε* is the lattice mismatch between the TiN nanocrystallites and SiCN interfaces. Since SiCN interfaces transform into crystalline structure, it is difficult to calculate the lattice mismatch between TiN and SiCN. If it is assumed that the lattice mismatch is between 3% and 4%, the hardness enhancement (*ΔH*_*2*_) can be estimated to be about 4.8 GPa–6.4 GPa according to Eq. (3), compared with SiCN.

Combined with the hardness increments calculated from the modulus-difference (*ΔH*_*1*_) and alternating-stress field (*ΔH*_*2*_) theories, a total hardness increment (*ΔH*_*1*_ + *ΔH*_*2*_) of 29.1 GPa–38.8 GPa can be achieved, relative to SiCN (14.2 GPa). Namely, the calculated hardness of the TiSiCN nanocomposite film from the coherent-interface strengthening mechanism is in the range of 43.3 GPa–53.0 GPa, which agrees well with the experimental value of 46.1 GPa. Therefore, the strengthening effect of the TiSiCN nanocomposite film can be well explained by the coherent-interface strengthening mechanism.

Under the epitaxial growth structure between the TiN nanocrystallites and SiCN interfaces, the generation and movement of dislocations can be effectively inhibited due to the stress field near the coherent interface^33, 34^, the sliding of the TiN nanocrystallite along the grain boundary can be also controlled owing to the epitaxial growth between the nanocrystallites and interfaces. Consequently, not only the mechanical properties of the film can be improved, but also the thermal stability is expected to be enhanced due to the fact that the grain growth can be restrained through the coherent structure.

## Conclusions

The quarternary TiSiCN nanocomposite films with the different C and Si contents are synthesized by reactive-magnetron-sputtering technique. The microstructure and mechanical properties of the TiSiCN nanocomposite films with the different C and Si contents are studied. The TiSiCN film is characterized as the nanocomposite structure with the TiN nanocrystallites surrounded by the (Si_3_N_4_ + C + CN_x_) interface phase. When the C/Si content ratio is 2:2, the TiSiCN nanocomposite film is remarkably strengthened with the maximal hardness and elastic modulus of 46.1 GPa and 425 GPa, respectively. Meanwhile, the (Si_3_N_4_ + C + CN_x_) interfaces do not present in the amorphous state, but exhibit as a crystallized form, which can coordinate the growth misorientations between the adjacent TiN nanocrystallites and maintain the coherently-epitaxial growth between the TiN nanocrystallites and interfaces. This investigation firstly provides the direct experimental evidence for the crystallized feature of the interfaces when the TiSiCN nanocomposite film is strengthened, suggesting that the strengthening effect of the TiSiCN nanocomposite film can be attributed to the coherent-interface strengthening mechanism, which is expressed as the “nc-TiN/c-Si_3_N_4_/c-C/c-CN_x_” model.

## Methods

### Film deposition

**Figure 7:**
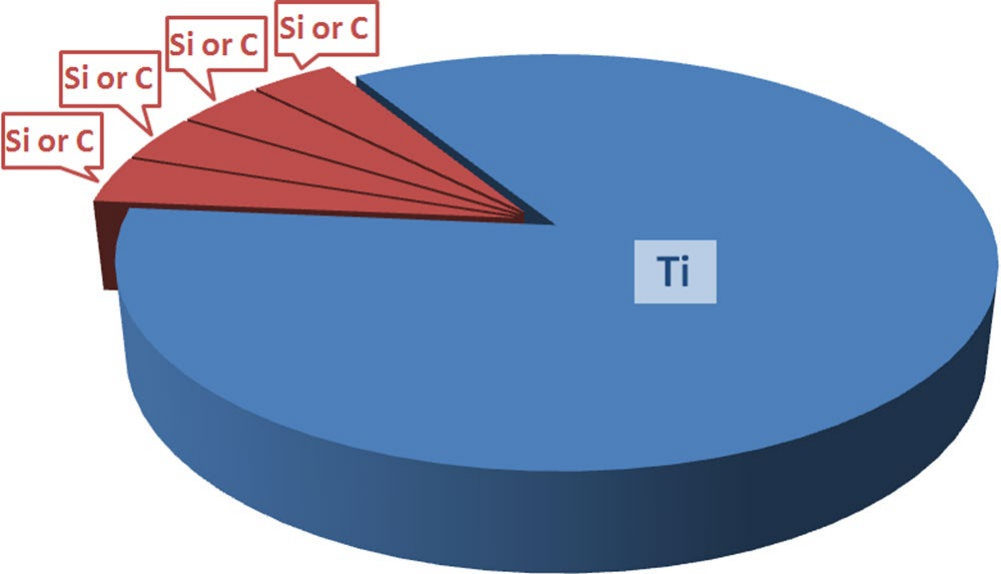
Schematic illustration of the TiSiC compound target used in the investigation. Through replacing different pieces of Si and C, the TiSiC compound targets with different Ti:Si:C volume or area ratios, including 21:4:0, 21:3:1, 21:2:2, 21:1:3 and 21:0:4, were prepared.

The TiSiCN nanocomposite films were fabricated on the silicon substrates by a JGP-450 magnetron-sputtering system. The TiSiCN films were sputtered from TiSiC compound targets (at.%, 99.99%) with 75 mm in diameter by the radio-frequency (RF) mode, and the power was set at 300 W. The TiSiC compound targets with different Si and C contents were prepared by respectively cutting the pure Ti (at.%, 99.99%), Si (at.%, 99.99%), and C targets (at.%, 99.99%) into 25 pieces. A total number of Si and C pieces was fixed at 4, and the number of the Ti piece was kept at 21 in this study in order to obtain the proper compositional ratio, which can be schematically illustrated in Fig. 7. Through replacing different pieces of Si and C, the TiSiC compound targets with different Ti:Si:C volume or area ratios, including 21:4:0, 21:3:1, 21:2:2, 21:1:3 and 21:0:4, were prepared. The base pressure was pumped down to 5.0 × 10^−4^ Pa before deposition. The Ar and N_2_ flow rates were 38 and 5 sccm (standard-state cubic centimeter per minute), respectively. The working pressure was 0.4 Pa, and the substrate was heated up to 573 K during deposition. To improve the homogeneity of films, the substrate was rotated at a speed of 10 r/min. The thickness of all the TiSiCN films was about 2 μm.

### Microstructural characterization

The microstructures of the TiSiCN nanocomposite films were characterized by XRD (Bruker, D8 Advance) with Cu K_α_ radiation (λ = 0.15406 nm) and field-emission HRTEM (FEI, Tecnai G2 F30) at an accelerating voltage of 300 kV. The grain sizes of the films were estimated by Scherrer formula^45^:

4 $${\rm{D}}=\frac{{\rm{0.9}}\lambda }{B\,\cos \,\theta }$$

where *D* is the grain size, *λ* is the wavelength of Cu K_α_, *B* is the full width of the half maximum (FWHM) of the measured peak, and *θ* is the typical diffraction-peak position. The composition of the film was characterized by an EDS accessory equipped in a scanning electron microscopy (SEM) (FEI, Quanta FEG450). The bonding structure was analyzed by XPS (Kratos AXIS Ultra DLD) with Al K_α_ (hν = 1486.6 eV) radiation source operated at 150 W. The preparation procedures of the cross-sectional specimen for the HRTEM observation are described as follows. The film with a Si substrate was cut into two pieces and adhered face to face, which was subsequently cut at the joint position to make a slice (Φ3 mm × 0.3 mm) (Gatan 601). The slice was carefully thinned by mechanical polishing (Gatan 623) and mechanical dimpling (Gatan 656), followed by low-angle argon-ion milling (Gatan 691).

### Mechanical tests

The mechanical properties (hardness and elastic modulus) were measured by a nanoindenter (Agilent, NANO Indenter G200) using the Oliver and Pharr method^46^. The measurements were performed by using a Berkovich diamond tip at a load of 5 mN with the strain rate at 0.05/s. At this load, the indentation depth was less than 1/10^th^ of the whole film thickness, thus minimizing the effect of the substrate on the measurements. Each hardness or elastic modulus value was an average of at least 16 measurements.
